# Mathematical Modeling of the Complement System: a Scoping Review

**DOI:** 10.1007/s11538-026-01723-2

**Published:** 2026-10-01

**Authors:** Luis Sordo Vieira, Daniel Cruz, Ivan Ramirez-Zuniga, Carl Atkinson, Borna Mehrad, Reinhard Laubenbacher

**Affiliations:** 1 Division of Pulmonary, Critical Care, and Sleep Medicine, Department of Medicine, University of Florida, Gainesville, USA; 2 Department of Mathematics, California Polytechnic State University, San Luis Obispo, USA; 3 Department of Mathematics, The University of Texas at Tyler, Tyler, USA; 4 Department of Epidemiology and Biostatistics, The University of Texas at Tyler, Tyler, USA; 5 Department of Surgery, Northwestern University, Chicago, USA

**Keywords:** Complement biology, Immunology, Mathematical modeling

## Abstract

The complement system is a potent arm of the immune system, linking the adaptive and innate immune systems, and affecting many facets of human pathophysiology. Recent advances in the development of complement-specific drugs in several rare diseases have opened the gateways to apply complement interventions toward a broader array of pathologies. However, this goal remains elusive due to the complexity and the multifaceted roles of the complement system. Mechanistic mathematical models can provide an effective tool to integrate the different mechanisms, features, and feedback loops into a framework that allows the simulation of complement dynamics under different conditions and the prediction of the effect of interventions. In this scoping review, we provide an overview of current mathematical models of the complement system, open problems related to complement biology, and discuss future work on how mechanistic mathematical modeling of the complement system can potentially alleviate some of the challenges in complement biology.

## Introduction to the Complement System

Jules Bordet discovered the complement system over a century ago (Bordet 1895; Cavaillon et al. 2019), when he noticed a heat-labile component of immunity that "complements the microbial clearance" by antibody-mediated immunity. The complement system consists of molecules that exist in quiescent form until a triggering event, such as infection or injury, rapidly activates them in an amplifying enzymatic cascade. Functionally, activation of the complement cascade results in the generation of molecules that enhance the recruitment and activation of immune cells, the ingestion of microbes by immune cells (phagocytosis), and direct killing of target cells by the membrane attack complex. Complement is most studied and best understood in the context of host defense against infections and inflammatory diseases, but recent evidence indicates that it also has a role in homeostatic mechanisms, such as the digestion of dead or dying cells (efferocytosis, (Hulsebus et al. 2016)) and tissue regeneration (Rutkowski et al. 2010). Due to its complexity, both in size and regulatory mechanisms, mathematical modeling has been used to attempt to better understand the systems dynamics and downstream effects of system activation in a systematic way.

**Fig. 1:**
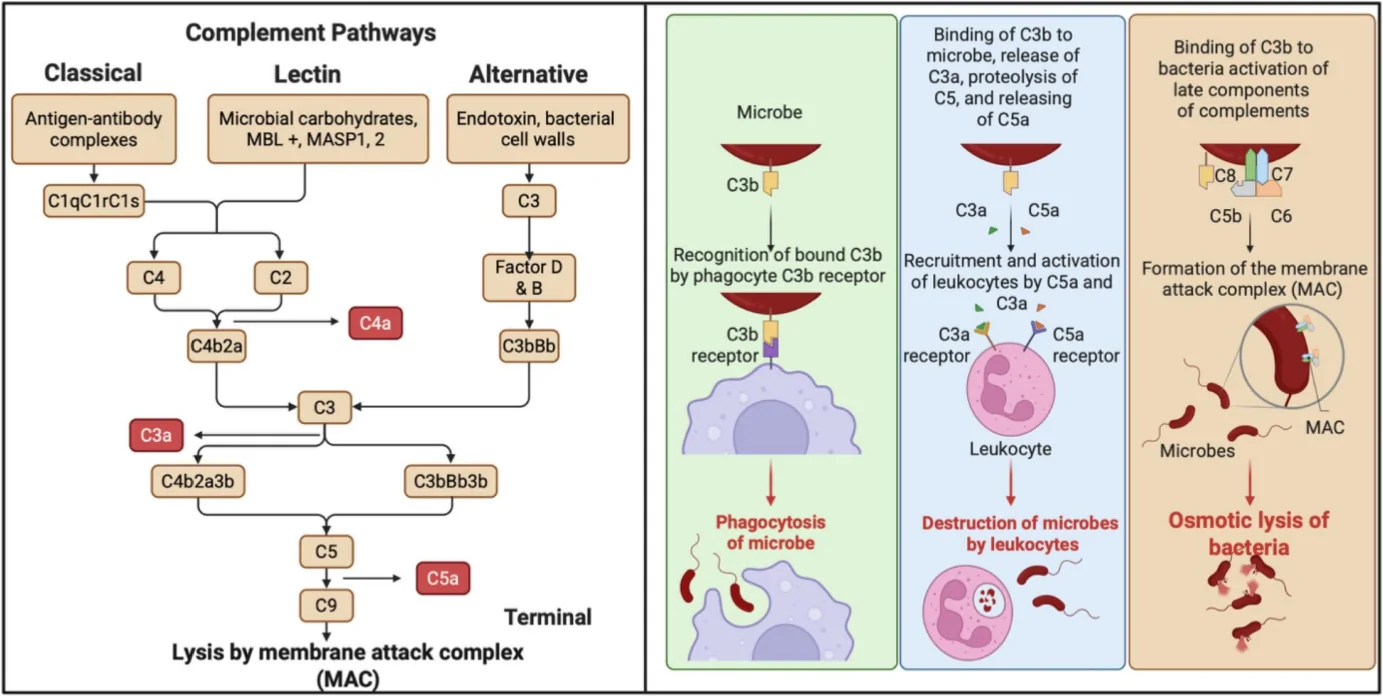
Complement activation is initiated by the classical, lectin, or alternative pathways, all resulting in C3 cleavage and terminal pathway activation (left panel). Right panel shows the functional outcomes of complement activation. Functionally, complement activation results in opsonization, stimulation of cells by the anaphylatoxins C3a and C5a, and formation of the membrane attack complex (MAC), leading to the lysis of pathogens. Figure adapted from Iwasaki, A. (2026) BioRender and Biorender Team (2026) BioRender. Created in BioRender. Sordo Vieira, L. (2026) https://BioRender.com/foqag01 (Color figure online)

The initiation of the complement cascade is categorized into three distinct pathways: the classical pathway, the lectin pathway, and the alternative pathway that converge on activation of complement factor C3 (Fig. 1). The first activating pathway to be discovered was the classical pathway, one of the main links of the humoral immune response to innate immunity. The first component of the classical pathway is C1q (part of the C1 complex, together with C1q, C1r, C1s), which binds antigen–antibody complexes and can also bind directly to the surface of pathogens. Once bound, this leads to a conformational change in the C1 complex, resulting in serine protease activity of C1s. The activated C1s cleaves C4 to produce two fragments, C4a and C4b, with C4b either rapidly covalently binding to a pathogen surface or quickly being inactivated by hydrolysis. C4b then binds a C2 molecule making it susceptible for cleaving by C1s, leading to the formation of the C3 convertase C4b2a. This C3 convertase can then cleave large numbers of C3 molecules, resulting in the generation of C3a anaphylatoxin and deposition of C3b on the pathogen surface which serves as an opsonin (a molecular tag on microbes enhancing recognition by immune cells).

The lectin pathway shares many similarities with the classical pathway, including a shared C3 convertase (C4b2b). In contrast with the classical pathway, however, the initiating event in the lectin pathway is binding the mannose-binding lectin (MBL), collectins, or ficolins to sugar moieties on invading pathogens. These pattern-recognition molecules are found to be associated with MBL-associated serine proteases (MASP), MASP-1 and MASP-2, which work in an analogous fashion to C1r and C1s, respectively. Ultimately, activating the lectin pathway leads to forming the C3 convertase C4b2b. Lastly, the alternative pathway is autoactivated by spontaneous hydrolyzation of C3, which constantly occurs at a low rate (called tickover). Hydrolyzed C3 can then bind to Factor B, and upon available Factor D, can be cleaved into the AP C3 convertase (called C3(H_2_O)Bb). Alternatively, cleaved C3b molecules bound to the surface of a pathogen can also bind further to factor B, leading to an amplification loop that encompasses approximately up to 80% (Harboe et al. 2004) of pathway activation.

Ultimately, all three pathways lead to the formation of a C3 convertase that cleaves C3 into the anaphylatoxin C3a and the opsonin C3b, which tags pathogens and dead cells for removal from tissues by phagocytes. This is then followed by a final common terminal pathway that culminates in the generation of the membrane attack complex (MAC), a pore-forming complex of C5b, C6, C7, C8, and C9 molecules, which kills target cells by permeabilizing them. Generation of MAC is downstream of C3b, which forms the enzyme C5 convertase by binding to a C3 convertase. C5 convertase cleaves C5 into C5a and C5b, initiating the cascade that terminates in the formation of the MAC on the cell membrane of pathogens (Fig. 1).

The anaphylatoxins, C3a and C5a, themselves play important roles in the immune responses. For example, C5a is a strong chemotactic factor for neutrophils and monocytes. It also induces endothelial cell activation, neutrophil extracellular trap formation, mast cell activation, and smooth muscle contraction. C3a, although traditionally described as a proinflammatory cytokine, is now known to also have anti-inflammatory effects, by, for example, inhibiting neutrophil mobilization (Wu et al. 2013; Coulthard and Woodruff 2015). Fragments of complement proteins also act as co-stimulatory molecules for the maturation of adaptive immune cells, providing a link between innate and adaptive immunity (Carroll 2000).

As complement is among the first lines of defense against pathogens, complement deficiencies are associated with various infections: Deficiencies in the C2 complement component are associated with recurrent infections by encapsulated bacteria (*e.g.*, *Streptococcus pneumoniae, Haemophilus influenzae, *etc*.*) (Figueroa and Densen 1991). Deficiencies in MAC components are associated with infections by bacteria of the *Neisseria* genus (Figueroa and Densen 1991). Pathogens have acquired a diverse set of mechanisms to hide from and inhibit the complement system (Nowacka et al. 2025). On the other end of the spectrum, complement dysregulation, particularly prolonged complement activation and/or overactivation, is associated with various pathologies, including cardiovascular disorders, kidney disease, cancer, and atypical hemolytic uremic syndrome. In addition to fighting infections, the complement system also plays an important role in preventing self-attack by the immune system (*e.g.,* clearing apoptotic cells). Accordingly, complement dysregulation is also associated with autoimmune disorders such as systemic lupus erythematosus and rheumatoid arthritis (Ballanti et al. 2013). Thus, the complement system must be tightly regulated to ensure that it quickly and effectively responds to insults; it is kept in check by a strong set of regulators on both the host’s cell membranes and in the fluid phase, which prevents self-attack. The short half-life of complement fragments further mitigates potential self-attack against the host.

In recent years, increased interest in the complement system has been sparked by the development of complement-specific therapeutics. For example, eculizumab—a C5 monoclonal antibody—was first FDA-approved in 2007 for paroxysmal nocturnal hemoglobinuria and has since been approved for atypical hemolytic uremic syndrome, generalized myasthenia gravis, and neuromyelitis optica spectrum disorder. More recently, a new drug (pozelimab-bbfg, an anti-C5 monoclonal antibody) by Regeneron Pharmaceuticals was approved as the first treatment for the rare genetic disorder, CD55 deficiency (also called CD55 deficiency with hyper-activation of complement, angiopathic thrombosis, and severe protein-losing enteropathy, or CHAPLE disease). As of late 2024, there are at least 12 FDA-approved complement-mediated therapeutics, targeting the pathway at different levels of the complement system, including for regulators, receptors of cleaved products, and inhibitors (Ricklin 2024; West et al. 2024; Padilla Kelley et al. 2025).

The complexity of the complement system makes it difficult to predict the effects of specific interventions, design optimal therapies, and untangle the complex dynamics that occur in multiple organs simultaneously. Mathematical modeling can alleviate some of these difficulties with appropriate data. Converting biological dynamics into explicit mathematical language can help researchers explore or assess the potential effects of certain interventions. Given the complexity of the complement system, some of the questions posed here are difficult to assess experimentally. Mathematical modeling can explain potential mechanisms that are consistent with given experimental data, prioritize targets, and identify most relevant mechanisms with tools such as sensitivity analysis, hence generating plausible hypotheses for experimental testing. Our goal in this manuscript is first to review existing mathematical models of complement biology and the results derived from these models. We then discuss a sample of open problems in complement biology for which mathematical modeling can help elucidate novel mechanisms. While computational models of complement continue to be developed (Zewde 2019), several avenues of research and challenges remain open (discussed in the sections below).

### Literature Search Method

We searched the manuscripts indexed by PubMed on July 23, 2024 using the search term (ordinary differential equations[tw] OR mathematical model[tw] OR computational model [tw] OR systems biology [tw]) AND (complement system[tw] OR complement cascade[tw] OR classical pathway [tw] OR lectin pathway [tw] OR alternative pathway [tw]), yielding an initial list of 61 publications. Among these, we selected the manuscripts specific to models associated with complement, and we then checked the bibliographies of those manuscripts to see if there were any referenced models that we missed. We also reviewed the bibliographies of all publications that had cited the manuscripts we had found, yielding a final count of 18 relevant manuscripts. One additional manuscript was found at the review stage. The section Questions Pertaining to Complement Biology with a Potential for Mathematical Modeling was generated based on reviewing the complement literature and authors’ research interests.

## Mathematical Modeling of the Complement System–An Overview

While several types of mathematical modeling frameworks have been used in systems biology (Machado et al. 2011; Le Novère 2015; Cruz and Kemp 2021), most models of the complement system exclusively involve ordinary differential equations (ODEs). These models are defined by sets of equations associated with target molecules; each equation describes the rate of change, with respect to time, of the concentration of one molecular species. As such, ODE models often generalize the location of the target molecules to compartments (*e.g*., cell surface, plasma) or ignore these locations altogether. Like several other types of mathematical models, ODE models often rely on parameters that have not been measured experimentally or collected within specific contexts (*e.g*., in vitro or in different mammalian models). These parameters are often estimated, calculated, or fixed so that the mathematical model agrees with a given data set. Each particular set of parameters is also chosen with certain assumptions; thus, a model’s outcome prediction should be considered under these same assumptions (within context). Given the spatiotemporal dynamics of the complement system, a few recent mathematical models have used other modeling paradigms (*e.g*., partial differential equations, logic-based rules) to mitigate the limitations of ODEs. We categorized complement biology models as compartmental or spatial models for the purpose of this review. The cited models are also briefly summarized in Table 1, including their goal and insight provided, limitations, and the extent to which uncertainty quantification is included in the manuscript.

**Table 1:** Summary of mathematical models of the complement system

Compartmental					
Reference	Main goals and key findings	Model type and description	Limitations	Model analysis/UQ	Model/Code/Data availability
Havsteen and Varón (1990)	Goal: To develop kinetic equations for the classical complement activation cascade under both transient-phase and steady-state conditions, and then derive the corresponding equations for rapid-equilibrium conditions. Additionally, proposed an experimental design for determining the kinetic parameters of the cascade from measurable progress curves Findings: The analysis showed distinct time behaviors among intermediates, including exponential approach to steady state and constant, linear, or parabolic behavior in steady state	ODE model 14 equations, 21 parameters) based on mass-action kinetics for theoretical analysis of CP	The paper studies a simplified fundamental cascade system and explicitly leaves out inactivation and inhibition steps in order to make the analysis tractable and to clarify the main principles of the kinetics. The authors also note that the practical usefulness of the theory depends on how many contributing species can actually be measured experimentally, since fewer observable species lead to more global/composite kinetic parameters rather than fully resolved fundamental constants	Includes a proposed experimental parameter-estimation strategy based on progress curves, induction periods, and related observables. No formal UQ framework	The model is available at the specification level in the paper itself: the reaction scheme, the system. No public repository is indicated
Hirayama et al. (1996)	Goal: To build a dynamical model of the classical pathway activation (using literature rate constants and serum concentrations), then linearize it and use linear systems analysis to characterize model behavior and how it changes under an optimization/feedback assumption Findings: Intermediate products in activation increased parabolically, while MAC increased linearly. Linear analysis showed activating reactions’ instability, mitigated by feedback control from MAC on initial activation	ODE model (26 equations, 22 parameters) based on mass-action kinetics for theoretical analysis of CP	Classical pathway only. Linear-systems results rely on a linearized approximation (inactive precursors treated as constant). The feedback/optimality assumptions aren’t experimentally supported, and they note more precise data are needed for validation	Parameters were taken from published sources, including literature-derived rate constants and previously reported serum concentrations and molecular weights. Includes a parameter-perturbation/robustness analysis. No formal UQ framework	Model equations and parameter tables included in appendix or main text. No public repository is indicated
Head et al. (1996)	Goal: To compare simulated behavior to reported immune-response kinetics, assess stability of the simulated immune-response system, and identify conditions that generate high immune-complex levels like those seen in immune-complex diseases Findings: Model mirrored concentration–time profiles, matching primary and secondary immune responses, long- and short-term immunity. Immune response perturbation led to oscillating intermediate concentrations, nonlinear antigen–antibody relationships, and exponential immune complex growth	ODE model (13 equations, 20 parameters) based on mass-action kinetics, of immune response including opsonization and clearance	Several complex sequences (specific cytokine-release cascades, antibody production, C3 convertase assembly) are represented as single rate-determining processes The system is treated as open (non-equilibrium) and governed by mass-action kinetics, with rate constants assumed independent of other reactions Only 8 of 19 rate constants were estimated from literature kinetics	Includes deterministic sensitivity/stability analysis by varying parameters ± tenfold, one at a time and in pairs, and assessing whether the system returns to steady state or becomes unstable. No formal UQ framework	Model equations and parameter included in main text. No public repository is indicated
Korotaevskiy et al. (2009)	Goal: To study nonlinear dynamics of the CP and AP using mass-action and Michaelis–Menten kinetics. Characterize quantitatively the background state of the complement system (CS) and study the dynamics of MAC formation Findings: Discovered a marked lag phase in CS activation. Deficiencies in individual proteins of CS have a limited effect on its overall physiological function	ODE model (33 equations, 54 parameters)	No target-cell/population growth equation, only fits cases where target growth is slow relative to complement activation Model assumes target cells have no membrane-bound complement regulators (e.g., DAF/MCP/CRRY), limiting relevance to many host-cell contexts	Includes parameter estimation via optimization. No formal UQ framework	Model equations and parameter tables included in appendix or main text. No public repository is indicated
Nypaver et al. (2010)	Goal: To use a simplified model to analyze luminescent *Klebsiella pneumoniae *in vitro data, assessing complement attack dynamics in healthy individuals and septic patients Findings: Work extends real-time technique for complement-mediated bacterial killing assessment. *K. pneumoniae* showed rapid C3 deposition; killing followed a lag. Opsonization began within 10 min, eradication took nearly 2 h. AP primarily responsible. Serum killed bacteria faster than β-lactams but slower than fluoroquinolones. Patients with sepsis showed accelerated activation rates, indicating complex immune responses	DDE model (3 equations, 7 parameters)	The dynamical model is intentionally lumped: classical/lectin/alternative pathway activation contributions in patient samples cannot be separated Bacterial strain used is not virulent and susceptible to complement-mediated killing Some parameters were empirically determined, while others were estimated via least squares	Includes sensitivity analysis and parameter fitting by nonlinear least squares. No formal UQ framework	Equations provided in the manuscript. No public repository is indicated
Liu et al. (2011)	Goal: To build a detailed mechanistic model of human complement regulation (activation + inhibition) and train/validate it against experiments, using a dynamic Bayesian network (DBN) probabilistic approximation to make parameter estimation and sensitivity analysis feasible for a large ODE system Findings: They used a DBN approximation to estimate parameters from experimentally generated test data, and validated the calibrated model using published bacterial killing rate observations Sensitivity analysis identified key mechanisms: the CRP–L-ficolin interaction and decay of the C3 convertase came out as crucial Computed antibacterial response curves vs pH and calcium, confirming prior findings and further elucidating how inflammation-like conditions enhance classical–lectin crosstalk	ODE model including classical, lectin, and amplification via CRP–L-ficolin crosstalk, with 42 species, 45 reactions, 85 kinetic parameters (71 unknown)	Large, highly parameterized ODE network; risk of practical non-identifiability and reliance on priors/literature values; DBN is used as an approximation partly to handle this complexity DBN is a probabilistic approximation of ODE dynamics; approximation quality can affect inference, especially for stiff/fast reactions Training/validation is based on specific in vitro assay conditions (pathway initiation via PC/GlcNAc beads; infection-inflammation–style perturbations; C4BP perturbation experiments). Generalization beyond these conditions needs caution	Includes Bayesian parameter estimation using a dynamic Bayesian network (DBN), together with sensitivity analysis and explicitly defined prior distributions for variables and parameters in the Supporting Information. No formal UQ framework	The model has been curated in BioModels (BIOMD0000000303, (Malik-Sheriff et al. 2020)) with SBML and auto-generated exports (including a Matlab file) Extensive Supporting Information provided(model description, parameter tables, priors/DBN structure)
Zewde et al. (2016)	Goal: To build a comprehensive quantitative dynamical model of the alternative pathway (across four stages: initiation, amplification, termination, and regulation) of complement to understand the balance between activation on pathogens and regulation on host cells under physiological conditions Findings: Model highlights the tight regulation of complement, inhibiting AP propagation by dismantling fluid phase convertases, while also emphasizing properdin’s role, released from neutrophils, in initiating and spreading the AP during bacterial infection	ODE model (107 equations, 74 parameters) Mechanistic ODE-based systems biology model of the complement alternative pathway	The model relies on many literature-based and assumed parameters, uses simplified host/pathogen surface representations, and adopts a mostly well-mixed (non-spatial) short-timescale framework, so it may miss important biological heterogeneity, transport effects, and longer-term immune interactions	Includes multiparametric sensitivity analysis. Limited direct experimental calibration/validation. No formal UQ framework	All relevant data are provided in the paper and Supporting Information, including kinetic constants, concentrations, sensitivity ranges, the full ODE system, and surface amplification details. No public repository is indicated
Sagar et al. (2017)	Goal: To understand the strengths and weaknesses of the complement cascade with a simplified model Findings: Complement was robust to any single therapeutic intervention. Simultaneous C3 and C5 knockdown effectively disrupts complement activation. Model predicted the necessity for multiple points of therapeutic intervention to fully disrupt complement activation	ODE model with logical rules with 18 Diff equations, 28 parameters Model describes the lectin and alternative pathway	No explicit classical pathway initiation (C1) and no MAC was included in the model Factor B and factor D assumed in excess C3a dynamics were too fast compared to the data	Includes parameter estimation from in vitro time-series measurements of C3a and C5a using Dynamic Optimization with Particle Swarms (DOPS), validation against unseen C3a and C5a measurements, global sensitivity analysis (Sobol), and robustness analysis. Does not present a full UQ framework	Code available at: https://github.com/varnerlab/Complement_model_repository
Zewde and Morikis (2018)	Goal: To develop a comprehensive computational model of the alternative and classical complement pathways to study complement regulation under homeostasis, Factor H (FH) impairment (disease/disorder state), and drug intervention (compstatin and eculizumab), and to evaluate biomarker responses across these conditions Findings: Factor H impairment pushes the complement system into a dysregulated state (depleting upstream components and increasing activation products), and that treatment effects depend on the inhibition target: compstatin (C3) works better for early-pathway biomarkers, while eculizumab (C5) works better for terminal-pathway biomarkers. The model also suggests combined targeting can help, but high doses may over-regulate the system	Mechanistic ODE-based complement systems model spanning both alternative and classical pathways with 290 ODEs and 142 kinetic parameters	Many parameters are obtained from literature or estimated/assumed when experimental values are unavailable, including borrowing kinetic rates from homologous/analogous proteins/interactions High structural/parametric complexity (290 ODEs, 142 parameters) increases risk of non-identifiability and compensation among parameters unless tightly calibrated to rich datasets	Includes multi-parametric sensitivity analysis (MPSA). No formal UQ framework	All relevant data are included in the article and its Supporting Information, including model equations and parameter tables. No public repository is indicated
Lang et al. (2019)	Goal: To develop a mathematical model of molecular crypsis in which *Candida albicans* acquires human factor H (FH) to reduce complement attack and become harder to distinguish from host cells Findings: Pathogen abundance relative to host abundance is the dominant factor controlling whether molecular crypsis succeeds, and that when pathogens gain a sufficiently strong quantitative advantage, autoreactivity can emerge	Evolutionary/game-theoretic + mechanistic ODE model (23 equations and 22 kinetic constants)	Model assumes a step function for attack, assumes infection with a single pathogen in one formula, and in the evolutionary model assumes that the C3b distribution is defined by FH acquisition alone. It also directly states that factors B, D, and I were fixed, C3a and iC3b were treated as sinks, and C3/FH inflow-outflow terms were chosen to recover physiological steady states	Includes sensitivity of the model with respect to assumptions about C3 and FH dynamics and explores alternative scenarios in the Supporting Information. No formal UQ framework	The model is available through the paper and supporting files, including parameter tables and the complete ODE formulation, and the article states that all relevant data are contained in those materials. No public repository is indicated
Bakshi et al. (2020)	Goal: To understand steady state response of AP activation and regulation including positive regulation of properdin with parsimonious ODE model Findings: Disrupting negative AP regulation leads to pathological dysfunction, while positive regulation elevates the baseline of C3 convertase. Negative regulation by FH-mediated regulation relies on FI-mediated. inactivation of C3b, which is countered by properdin, protecting C3b from inactivation	Developed two ODE models of the AP referred as the minimal model (9 equations, 22 parameters) and the properdin model (14 equations, 31 parameters)	All parameters were taken from the literature but the model was not fitted to experimental data The model is fluid-phase only, so it does not capture surface-bound/local complement activation or the effects of surface regulators, meaning conclusions (especially about properdin) may not fully transfer to tissue- or cell-surface contexts Modeling assumption limitation: it treats the system as well-mixed with mass-action kinetics; for surface activation this can be inappropriate because reactions can be highly local/spatially inhomogeneous	Includes relative sensitivity analysis for minimal and properdin models, together with nondimensionalization No formal UQ framework	Model equations and parameter tables included in appendix or main text. MATLAB code reported as available in the Supplementary Material; not identified in the files accessed. However, the MATLAB code is available via BioModels (BIOMD0000001016-18) (Malik-Sheriff et al. 2020)
Caruso et al. (2020)	Goal: To build a single mechanistic mathematical model of the alternative complement pathway (AP) that can explain both in vitro complement assays and in vivo behavior in health and disease, especially paroxysmal nocturnal hemoglobinuria (PNH), and to use that model to study complement-driven hemolysis and the effects of eculizumab treatment Findings: The model provided an accurate description of pathway activity in vitro and in vivo and could reproduce an array of standard assays	ODE model (226 equations, 121 parameters) of AP adapted from Zewde et al. (2016). Model links complement activation to hemolytic activity to reproduce experimental in vivo and in vitro observations	Model captures much of the biology but still misses some quantitative details Model relies heavily on data aggregated from many published experiments performed under different conditions, with some data digitized from figures	Includes parameter fitting and local sensitivity analysis with respect to the fitting objective and terminal pathway activation. No formal UQ framework	All relevant data are within the manuscript and its Supporting Information files Code available at: https://github.com/denhardt-lyox/alternative-complement-pathway
Zewde et al. (2021)	Goal: To build a comprehensive predictive model of the complement system that spans all three pathways and use the model to compare two pathogen types and to study how environments such as the bloodstream or nasal cavity alter complement response at the level of opsonization or MAC deposition Findings: In the model, both pathogen types in the bloodstream become highly occupied by complement proteins, while MAC occupies much less than 1% of the pathogen surface	ODE model (670 equations, 328 parameters) of CP, AP, and LP, incorporating the effects of a complement susceptible pathogen and Neisseria meningitidis	Findings are exclusively computational and were not supported or refuted by new experiments With an elevated number of equations and unknown parameters, the model is very likely to face serious practical identifiability issues if one tried to estimate many parameters from data	Includes global sensitivity analysis using multiparametric sensitivity analysis (MPSA), Latin hypercube sampling, 1,585 kinetic variations, and a Kolmogorov–Smirnov statistic. No formal UQ framework	Summary of variables, reactions, parameter values/sources, and ODEs is provided in supplementary files No public repository is indicated
Bansal et al. (2022)	Goal: To build a quantitative systems pharmacology (QSP) ODE model of human complement activation focused on the alternative + terminal pathways Use the model to evaluate and prioritize drug targets (including some short-lived intermediates) for complement-driven autoimmune disease mechanisms and to estimate “dosing tractability” for small molecules vs large-molecule antibodies Findings: Inhibiting complement proteins reduces activation and lysis. Challenges in engaging high-concentration proteins with large-molecule antibodies. Small-molecule compounds face affinity challenges with short-half-life fragments. Disease severity reduction requires higher affinities than conventional target engagement. The model stresses the importance of accounting for target engagement in lead optimization stage	ODE model (311 equations, 139 parameters) of complement activation via the AP including dynamics of some regulatory proteins, including properdin	Many kinetic parameters come from literature, but when there is no direct literature evidence, parameter values were assumed from similar processes/proteins The model is framed as activation “in plasma and on the surface of erythrocytes,” which implicitly excludes other tissues, organs, and relevant host-cell surfaces The model considers the lysis of red blood cells (RBCs) only, while diseases like atypical hemolytic uremic syndrome (aHUS) involve other host cells Peptides were not assessed, and they considered systemic dosing only, noting localized dosing might make some targets more feasible in specific tissues	Includes calibration/validation against multiple datasets. No formal UQ framework	A summary of the variables, reactions, parameter values and sources, and the SimBiology code used to implement the ODE system is provided in the supplementary files. Data is available upon request
Murad et al. (2023)	Goal: To construct a qualitative computational model of SARS-CoV-2–induced complement signaling pathways and use it to study how signaling patterns differ between normal recovery, pathogenic hyper-inflammation, and intervention scenarios aimed at managing overactivation. The paper is especially focused on the roles of C3, C5a, and pro-inflammatory cytokines in shaping immune-system behavior during infection Findings: Under normal signaling, pathogen-triggered activation leads to recovery, whereas under pathogenic signaling, persistent infection drives the system toward a hyper-inflammatory stable state. In both cases, homeostatic oscillations represented by strongly connected components were observed, but the pathogenic case additionally showed recurrent infection, instability of the normal state, and a deadlock state. In the intervention cases, the model removed the deadlock state and redirected trajectories toward homeostasis or a normal immune response, although recurrent infection could still appear in some parts of the state graph	A qualitative, discrete model of SARS-CoV-2–induced complement-system signaling. The model is built as a biological regulatory network (BRN) using the René Thomas asynchronous multi-valued kinetic logic framework	The model is qualitative and discrete, so it does not estimate continuous concentrations, kinetic rates, or time-resolved quantitative trajectories. Its parameters are logical parameter sets chosen to satisfy Computation Tree Logic (CTL) constraints rather than biophysical rate constants. Because of that, the model is better suited to exploring possible signaling patterns and attractors than to making quantitative predictions about magnitudes or timescales	Includes constraint-based parameter selection via model checking to identify parameter sets that satisfy CTL specifications derived from experimental observations. No formal UQ framework	The model code, SMBioNet model-checking algorithm and associated code, logical parameter sets, and biological regulatory network (BRN) and state-graph files rendered in Cytoscape are available in the Supplementary Files
Reeve (2024)	Goal: To use a revised in silico mass-action model of the AP of complement to study how pulmonary COVID-19 infection can perturb innate immunity through lectin-pathway stimulation, Factor H inhibition, and erratic viraemia. It focuses on the first 10 days of infection, before adaptive immunity develops, and asks how host-to-host variation in complement protein levels and different viral release patterns affect C3 convertase dynamics, viral elimination capacity, and the risk of C3 exhaustion Findings: the AP can generate C3 convertase responses spanning roughly three orders of magnitude, enough in some scenarios to track viral growth, but that this wide response range comes with a loss of stability because the system can undergo near-complete and prolonged C3 exhaustion. Exhaustion is more likely when viraemia rises in step-like bursts than when it increases smoothly, and it is further amplified by conditions such as type 2 diabetes, hypertension, short-term viraemia variability, pulmonary capillary clotting, and secondary acidosis. The central biological conclusion is that the AP’s positive feedback greatly expands its response range at the expense of robustness, making innate immunity vulnerable to destabilization by certain viral time courses and host conditions	Revised steady-state algebraic Mass Action model	On the modeling side, it considers only the upstream alternative pathway through C3 convertase, excludes downstream complement effects, uses simplified assumptions for viral growth/release and some biochemical interactions, and approximates the dynamics with difference equations, which become less accurate when C3 levels are very low. On the biological side, several key quantities had to be estimated from limited data, including using sparse fibroblast data for regulation of C3 and factor B synthesis even though the liver is the main production site, and assuming C3a-driven feedback, possibly via IL-6, regulates C3 and FB up-regulation despite uncertainty about how strong or saturating that effect is. Overall, the authors indicate that better data on COVID-19 viraemia bursts and on C3/FB regulation during infection would likely require updating the model	Includes host-variability and scenario-based sensitivity analysis, with starting protein concentrations randomized from published normal ranges and the model evaluated under alternative viral-growth and pathophysiologic scenarios. No formal UQ framework	The model is available at the specification level in the paper: the article gives the updated mass-action framework, the core equations, and complement protein concentrations and rate determinants used in the model. Model implemented in JMP 13. The paper includes an explicit statement that the dataset is available through the Dryad Digital Repository

Spatial					
Kalita et al. (2006)	Goal: To build a software system that models immune-system participants at the cell level, simulates their interactions, and helps infer overall system behavior in normal and simple disease situations. A secondary goal is to provide a flexible platform that can later be extended to more detailed disease simulations, including a simple anthrax infection scenario Findings: SIMISYS can qualitatively reproduce several expected immune-response patterns across four simple infection scenarios. In milder scenarios, neutrophils appear early and are sufficient to control infection. In more severe scenarios, macrophages, then dendritic cells, and later B cells/antibodies become involved, with delays before bacterial control is achieved	Cellular automata model to model innate and adaptive immune responses against bacterial infection with a graphical user interface. The model includes both innate and adaptive components, including macrophages, dendritic cells, neutrophils, NK cells, B cells, T helper cells, complement proteins, antibodies, and a generic bacterium	The model includes only a subset of immune-system chemicals, it uses a relatively small simulated cell population compared with the true immune system, assumes only one generic bacterium in the main version, and simplifies many cell behaviors into rule-based interactions. The authors also describe the model as a proof-of-concept that is being continuously improved The paper is primarily a software/platform paper rather than a tightly validated biological modeling study. The results are demonstrated through a small number of hand-designed scenarios, and the authors mostly judge success by whether the behavior seems to match “real life” qualitatively	Includes user-specified model inputs such as initial counts, lifespans, grid size, diffusion constants, and breakdown constants, but does not describe parameter fitting to biological data or statistical estimation. No formal UQ framework	No formal public code or data availability statement in the paper
Conrad et al. (2013)	Goal: To develop a spatial reaction–diffusion model to study how complement activation, especially C5a production, occurs within the extracellular polymeric substance (EPS) surrounding *Staphylococcus epidermidis*. The main biological question is how EPS thickness changes the kinetics of complement attack and the likelihood that nearby host cells detect the bacterium through C5a signaling Findings: For a single bacterium, the model predicts that C3 diffuses into the EPS very rapidly, reaching equilibrium with plasma in about 10 ms, while the buildup of active C3/C5 convertase is much slower and peaks around 15 min. The paper also finds that including diffusion is important: if diffusion is omitted, the amount of active convertase is dramatically underestimated. In addition, the sensitivity analysis indicates that EPS thickness and density of EPS binding sites matter more for C5a production than do the diffusion coefficients themselves	PDE reaction diffusion model (5 equations and 8 parameters)	It is a simplified computational model built with several assumptions and with parameter values that do not fully match the exact biological system. In particular, some key inputs were not directly available for *S. epidermidis*: the paper says diffusion coefficients in biofilm were not available and were therefore estimated from dextran measurements, and the discussion states that no direct data were available for the concentration of complement C3 binding sites in *S. epidermidis* EPS	Includes time-dependent normalized sensitivity analysis to assess how model parameters influence outputs such as C5a concentration over time. No formal UQ framework	The supplement provides the model specification, and the study relies mainly on published data and literature-based parameters, but there is no explicit public code or standalone dataset provided in the uploaded materials
Tille et al. (2020)	Goal: To develop and analyze a hybrid ODE–PDE model of complement dynamics, called DynaCoSys, to quantify how Factor H controls self vs. non-self discrimination at cell surfaces. In particular, the model is used to predict when Factor H binding is sufficient to suppress opsonization and permit complement evasion, and to identify which processes and parameters most strongly affect the threshold between self-tolerance, non-self recognition, and an intermediate evasion regime Findings: The model identifies three qualitative regimes as surface Factor H increases: a non-self-recognition regime with strong opsonization, a self-regime with strong suppression of opsonization, and an intermediate complement evasion regime	Hybrid model (5 equations, 35 parameters) with ODEs for cell-bound molecules, PDEs for fluid phase concentration profiles	Model is a reduced representation of complement dynamics, it relies heavily on effective rates and assumptions, and it is constrained by limited quantitative data, especially for time-resolved surface-bound molecules. The model focuses mainly on C3-derived species, lumps many other complement molecules into effective processes, assumes some fluid-phase concentrations are effectively constant, and uses quasi-steady-state approximations (QSSA) for fast processes	Includes local sensitivity analysis. No formal UQ framework	The paper provides the full model in the Supplementary Material, including the reaction network, ODE–PDE structure, and model reductions. No public repository is indicated

Classical Pathway (CP), Alternative Pathway (AP), Lectin Pathway (LP). Dynamic Bayesian Network (DBN). Extracellular polymeric substance (EPS). (ODE) Ordinary differential equations. (PDE) Partial differential equations, (DDE) Delay Differential equations, acronyms RBCs (red blood cells), aHUS (atypical hemolytic uremic syndrome), CS (Complement System) and UQ (Uncertainty Quantification)

### Compartmental Models

Havsteen and Varón formulated the earliest model to focus on complement in 1990 (Havsteen and Varón 1990), composed of 14 equations, focused on a theoretical basis for analyzing the classical pathway. The use of arbitrary rate constants and initial conditions limits this model. To address this issue, Hirayama et al. developed the first model to employ experimentally derived parameters (Hirayama et al. 1996). This model is still small (in comparison to later models) and covers the classical and terminal pathways; however, it assumes that its 26 equations can be approximated with linear equations to simplify the analysis. It is worth noting that both of these early models employed mass action kinetics, while subsequent models included Michaelis–Menten kinetics as well.

**Fig. 2:**
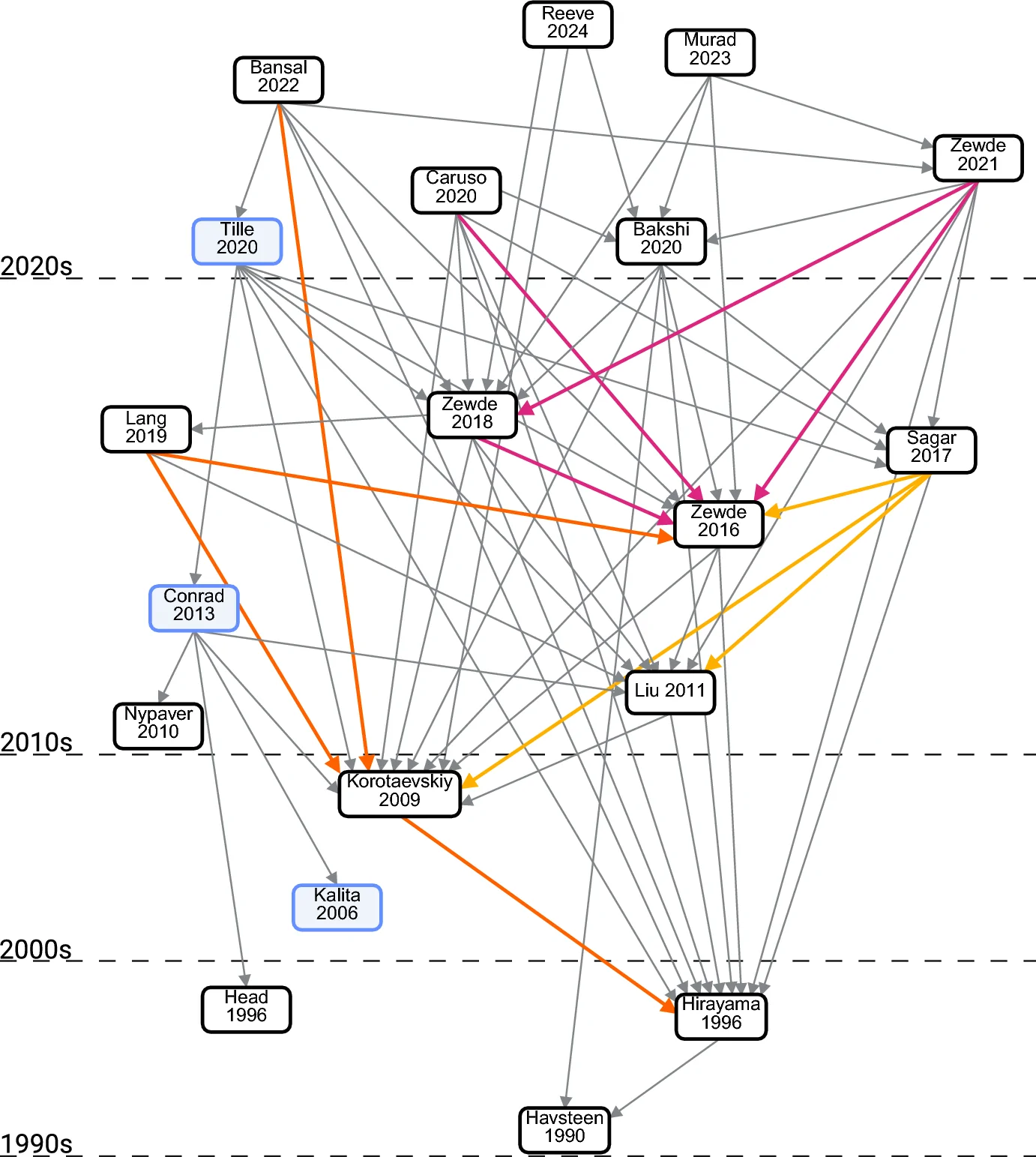
A graphical representation of the evolution of mathematical modeling of complement. Incoming arrows represent citations. The coloring on the arrows represents the following: magenta—model expan- sion, yellow—model simplification, orange—model modification, and light gray—none of the above. Blue shaded boxes represent spatial models. Color figure available online. Figure created in BioRender. Sordo Vieira, L. ( 2026) https://BioRender.com/1nn9lkx

Expanding on this work, three mathematical models of increasing size by Korotaevskiy et al. (2009), Liu et al. (2011), and Zewde et al. (2016) stand out as foundational. Every subsequent model of complement cites at least one of these models, often aiming to improve and/or broaden the referenced work (Fig. 2). The ODE model by Korotaevskiy et al. was specifically constructed to remove the linearity assumption of the model by Hirayama et al. and focuses on the dynamics of the alternative, classical, and terminal pathways. The authors used this model to explain how pathogen destruction is affected by the regulation of MAC formation (Korotaevskiy et al. 2009). The ODE model by Liu et al. focuses on specific up- and down-regulation mechanisms in the classical and lectin pathways and does not explicitly capture the alternative pathway; the authors note how these mechanisms may be targeted for future therapies (Liu et al. 2011). On the other hand, the large ODE model (107 equations) by Zewde et al. provides an extensive description of the alternative pathway, covering initiation, amplification, termination (via MAC), and regulation. To this end, the model was tested in the context of both pathogen and host cell surfaces (Zewde et al. 2016). A key feature shared by all three of these fundamental models is that mathematical techniques were used to determine several parameters: (i) Korotaevskiy et al. used an optimization procedure (Korotaevskiy et al. 2009), (ii) Liu et al. used dynamic Bayesian network approximation for both parameter estimation and sensitivity analysis (Liu et al. 2011), and (iii) Zewde et al. used various approaches to calculate, estimate, or optimize many of the rate constants in their model (Zewde et al. 2016). While a few of the later ODE models we include in this review address these limitations by predominantly using experimentally derived parameters (Sagar et al. 2017; Bakshi et al. 2020; Caruso et al. 2020; Bansal et al. 2022), most subsequent models have inherited this limitation.

In a broad sense, we can divide the mathematical models referencing the work by Korotaevskiy et al., Liu et al., and Zewde et al. into two categories: “small” (*i.e., *reduced order) models (Sagar et al. 2017; Lang et al. 2019; Bakshi et al. 2020; Reeve 2024) and “large” models built upon at least one of the foundational models above (Zewde and Morikis 2018; Caruso et al. 2020; Zewde et al. 2021; Bansal et al. 2022). Models in the first category have parameters that are primarily derived from experiments; most are also validated against experimental literature. The first of these “small” models is a hybrid ODE model of the alternative and lectin pathways that incorporates logic-based rules. This model was designed to have fewer equations than the three foundational models above and to compare equally or favorably to these models in experimental validations (Sagar et al. 2017). The ODE model by Lang et al. is directly adapted (and reduced) from (Korotaevskiy et al. 2009) and (Zewde et al. 2016) to study molecular crypsis. This model was applied to predict C3b opsonization for both *Candida albicans* and *Escherichia coli*, thereby providing insight into how *Candida albicans* could use Factor H (an inhibitor of C3 activity) to “hide” from complement (Lang et al. 2019). Next, there are the two parsimonious ODE models by Bakshi et al. of the alternative pathway in the fluid phase, the smaller of which (9 equations) considers steady-state response and the larger of which (14 equations) incorporates positive regulation via properdin, the only known positive regulator of the alternative pathway. These models were designed to capture the minimal machinery needed to represent the alternative pathway at a steady state and are implemented with almost completely experimentally derived parameters (Bakshi et al. 2020). Finally, there is a mass action model (4 equations) of the alternative pathway by Reeve which partially builds off the models by Bakshi et al. This model focuses on simulating the complement system’s response to a COVID-19 infection under different conditions corresponding to plausible variations in humans. This model is notable in that its development involved the generation of cohorts of “simulated” patients based on available clinical and experimental data (Reeve 2024).

We consider the four mathematical models in the second category to be “large” because they each have over 200 equations. Two of these (Zewde and Morikis 2018; Zewde et al. 2021) are sequential expansions of each other (Zewde et al. 2016). The 2018 model extends the mathematical description of the alternative pathway, includes the terminal pathway, and captures the fluid phase activation and propagation of the classical pathway (Zewde and Morikis 2018). The 2021 model is the largest mathematical model of complement to date, with over 600 equations and over 300 parameters; the authors developed this model with the goal of having it serve as a comprehensive representation of all four pathways (classical, lectin, alternative, and terminal) (Zewde et al. 2021). The ODE model by Caruso et al. is also based on the 2016 model by Zewde, including a description of the terminal pathway to study the effects of the alternative pathway on hemolysis and the role of this pathway as a driver of disease. As part of their interest in studying complement dysregulation, the authors incorporated a mathematical description of anti-C5 antibody eculizumab within their model. They compared their model predictions of applying this pharmacological intervention with clinical observations (Caruso et al. 2020). On the other hand, the ODE model of the alternative and terminal pathways by Bansal et al. was only partially built from the model by Korotaevskiy et al., with several equations based on mass action kinetics and original in vitro data from assays. This model was crafted to describe complement over-activation, evaluate certain complement proteins as potential drug targets, and evaluate drug dosing requirements for humans (Bansal et al. 2022). We note the models by Caruso et al. and Bansal et al. employed several parameters that were estimated, calculated, or otherwise inherited from the calculations and/or estimates of other models; however, the authors of these models also validated their results against in vitro, in vivo, and/or clinical data in order to mitigate any issues that might arise from using such parameters (Caruso et al. 2020; Bansal et al. 2022).

We conclude our discussion of non-spatial models by commenting on those which describe complement at a high level. These mathematical models often make simplifying assumptions and abstract several biological interactions. The earliest example is a general-purpose model by Head et al. of immune complex formation, opsonization, and clearance. In this ODE model, complement is described in the context of opsonization with just one variable (and thus one equation) (Head et al. 1996). The delay differential equations (DDE) model by Nypaver et al. (Nypaver et al. 2010) involves a slightly less abstract description of complement: the three initial pathways (alternative, classical, and lectin) are represented by coefficients in one equation with the purpose of representing the activation of complement. This model was developed for a specific context, the complement-mediated killing of *K. pneumoniae*, and mainly served as a basis for statistical analysis against in vitro data (Nypaver et al. 2010). The most recent collection of high-level models of complement found in Murad et al. (2023) differs from these two earlier models in that: (i) the models use multi-valued logic equations instead of ODEs, (ii) the models are distinguished from one another by their underlying biological assumptions, and (iii) each model was formulated and validated with in vitro and in vivo studies. The authors of these models used them jointly to study the innate immune response, particularly complement overactivation, during a SARS-CoV-2 infection and to suggest interventions that could control the pro-inflammatory cytokines’ expression levels in the associated cytokine storm (Murad et al. 2023).

### Spatial Models

To our knowledge, only three computational models have an explicit spatial component: an early agent-based model (Kalita et al. 2006) and two differential equation models (Conrad et al. 2013; Tille et al. 2020). The first model by Kalita et al. is a tissue-level model of immune response to a generic bacterial infection which includes several immune response cells (*e.g*., macrophages, neutrophils, and natural killer cells). This model focuses on the alternative, lectin, and terminal pathways. It uses a constantly generated and randomly distributed pool of complement proteins in the environment to tag pathogens and eventually pierce them via MACs (Kalita et al. 2006). In contrast, the partial differential equation (PDE) models by Conrad et al. are the first mathematical models to explicitly represent the diffusion of complement components in a biological environment (*i.e.*, a single cell or a capillary). The authors developed these small models (5 equations per model) to estimate the timing and intensity of opsonization of *Staphylococcus epidermidis*. The larger model by Tille et al. goes one step further by utilizing ODEs and PDEs to represent interactions involving surface-bound and fluid phase molecules. This model primarily focuses on C3b and includes the activation, opsonization, stabilization, amplification, and regulation of the alternative pathway via C3 and its derivatives. The authors use their model to explore complement regulation and evasion via Factor H, specifically considering how this protein determines both self (host) and non-self (foreign) cells.

## Questions Pertaining to Complement Biology with a Potential for Mathematical Modeling

The evolving field of complement biology has led to many questions for which mechanistic mathematical modeling address some of the experimental challenges and lead to novel hypotheses of mechanisms. We present a non-exhaustive list of such questions.

### Local Versus Hepatic Biosynthesis of Complement Precursors

Historically, the main components of the complement system were thought to be synthesized exclusively by hepatocytes (Alper et al. 1969; Morgan and Gasque 1997). It is now well-established that complement components (*e.g*., C3, C1q, properdin, and factor D) can be made by various non-hepatic cell types such as epithelial cells, granulocytes, monocytes, and macrophages (Morgan and Gasque 1997). With advances in new sequencing technologies (*e.g*., scRNA seq and spatial transcriptomics), new sources of extrahepatic complement biosynthesis continue to be discovered. (Chaudhary et al. 2022). For example, epithelial cells are an important source of complement proteins in the lung (Strunk et al. 1988; Kulkarni et al. 2019; Sahu et al. 2023), and a recent study showed that locally sourced C3 from lung epithelial cells plays a crucial role in providing protection against bacterial pneumonia (Sahu et al. 2023). Mouse biotechnology tools such as the Cre/lox and tamoxifen-induced Cre systems should further unravel not only the source of extrahepatic complement proteins but also the mechanistic role of extrahepatic protein biosynthesis. Several questions remain regarding the local vs hepatic biosynthesis of complement proteins. One possibility is that local complement biosynthesis might be more influential at serum-restricted sites, potentially serving as an initiator followed by a pulse of serum-derived complement due to the increase in vascular permeability from (local) complement activation (Lubbers et al. 2017). It is also suggested that some locally synthesized complement components might differ structurally and/or be post-translationally modified compared to the complement proteins found in the serum (West et al. 2020; Kremlitzka et al. 2022; West and Kemper 2023). Mathematical tools such as compartmental (*e.g*., ODE) and multiscale (*e.g*., agent-based) models could be used to explore the importance of proximity of complement proteins during insults, taking into consideration their rapid turnover, their short half-lives, and differences in abundance of inhibitors and regulators in local tissue. As an example, using an ABM model of a local insult to the alveolar space, one could compare the outcome of a bacterial infection with or without secretion of local C3 by alveolar epithelial cells, or by manipulating the abundance and speed of the pulse of liver-derived C3. Spatial proteomics/transcriptomics and immune fluorescence can help calibrate and validate such models, although currently such datasets remain largely scarce in the complement field. Importantly, current therapeutics target complement activation systemically, and little is known about how systemic inhibition affects local complement activation. Another example could be a PK/PD approach modeling how systemic administration of a therapeutic affects its local availability and, consequently, the local dynamics.

### Non-Canonical Activation and Inactivation of the Complement System

In addition to the canonical modes of activation of the complement system (*i.e*., activation via the classical, lectin, and alternative pathways), additional non-canonical modes of complement activation are now widely recognized, and others are under investigation. Amongst the most recognized is the crosstalk between the coagulation and complement cascades (Amara et al. 2008, 2010). Similar to the complement cascade, the coagulation system consists of a series of plasma proteins in inactive form that, once activated, generate a chain reaction of amplifying activation of successive reactions in which each coagulation factor goes from inactive to active after being cleaved by other coagulation factors. These reactions ultimately lead to the cleaving of prothrombin into thrombin, which converts fibrinogen into fibrin, stabilizing platelet aggregates. Several lines of evidence suggest that the coagulation and complement pathways may interact, bypassing canonical activation systems through shared activators and inhibitors. For example, thrombin—the key mediator of conversion of fibrinogen to fibrin—was found to act as a C5 convertase in the absence of C3 (Huber-Lang et al. 2006; Amara et al. 2008, 2010). It was later shown that thrombin can lead to different C5 cleavage products that support the generation of MAC (Krisinger et al. 2012). Plasmin—the central molecule in the fibrinolytic cascade (breakdown of clots)—can inhibit complement at the level of C3b and C5 (Barthel et al. 2012); this has been suggested as a mechanism by which pathogens evade the immune system (Barthel et al. 2012). Several studies also show that free extracellular heme (from, *e.g*., local hemolysis) can influence complement activation (Roumenina et al. 2011; Gerogianni et al. 2022). Overall, the complement and coagulation cascades appear linked at multiple levels, but the associated mechanisms are poorly understood. Linking the many mathematical models of coagulation (Hockin et al. 2002; Fogelson and Tania 2005; Link et al. 2020; Bannish and Hudson 2021; Owen et al. 2024) with new and existing models of complement could help clarify some of the molecular links between these two pathways. As these pathways directly interact and can either promote or inhibit one another, understanding the levels of their constituents and shared inhibitors that lead to equilibrium, as well as how dysregulation of one pathway can affect the other in disease, is a mathematically tangible problem that may lead to novel therapeutic strategies. Future questions regarding synergistic approaches targeting complement and coagulation can be facilitated by mathematical modeling—by, for example, prioritizing computationally predicted therapeutics.

### The Complosome

The effects of complement are traditionally thought to occur in the extracellular space. Evidence now suggests that active intracellular complement components (*i.e.*, the *complosome*) play important roles in various facets of homeostasis, metabolism, and immunity in various cell types (Liszewski et al. 2017; West and Kemper 2023). Nonetheless, some controversy still remains within the field about the role of intracellular complement and the intracellular location of these complement proteins. Intracellular complement proteins have been shown to interact with other intracellular machinery, including inflammasomes, lysosomes, and mitochondria (West and Kemper 2023). Interestingly, the core components of complement (C3 and C5) are not the only ones that play an important role in regulating cell behavior. Intracellular anaphylatoxin receptor signaling can mediate the activation of other intracellular machinery that controls growth, metabolism, cell survival, and immunity (*e.g.*, mTOR) (Hess and Kemper 2016; Ye et al. 2019; West and Kemper 2023).

Emerging research suggests that dysregulated complosome activity or expression of levels of intracellular complement proteins is associated with various pathologies, including diabetes, COVID-19, Crohn’s disease, and cancer (West and Kemper 2023). Several of the complement-specific therapeutics on the market (*e.g*., eculizumab, pegcetacoplan, or C1 esterase inhibitor) target only extracellular complement, leaving room for the discovery of novel intracellular drug targets. With advances in technology such as single-cell sequencing and especially spatial transcriptomics, combining systems biology approaches and mathematical modeling could extricate the contributions of intracellular complement signaling, autocrine complement signaling, and extracellular complement activity. Multiscale and multicompartment mathematical models of the complosome could also help untangle the multiple ways in which intracellular complement affects other crucial pathways and explain how it drives dysregulated behavior at the intercellular and tissue scales. For example, an ODE model linking intracellular signaling to metabolism could estimate thresholds for intracellular C3/C5 cleavage or receptor signaling that push a T cell, macrophage, or stem/progenitor cell from quiescence into an activated metabolic state (West and Kemper 2023). Due to the rapid dynamics of complement activation and the stochasticity of biological systems, designing experiments to assess such thresholds without model-based guidance would be inefficient.

### Complement in Immunometabolism

Complement activation products, such as C1q, C3a and C5a, can modulate cellular metabolism by altering the activity of enzymes involved in glycolysis, fatty acid metabolism, and oxidative phosphorylation. For instance, complement activation can impact the metabolic status of immune cells, inducing oxidative bursts on macrophages (Murakami et al. 1993; Merle et al. 2015). Interferon-gamma (IFN-γ) promotes the polarization of macrophages towards a pro-inflammatory phenotype, characterized by enhanced glycolysis and oxidative metabolism to support their effector functions, including phagocytosis and cytokine production. Additionally, IFN-γ can upregulate the expression of complement receptors on macrophages (Drevets et al. 1996) (especially in bacterial infections), thereby enhancing their responsiveness to complement activation products like C3a and C5a.

Mathematical modeling offers a promising approach to elucidate the complex dynamics and interactions between the complement system and metabolism during infection. Computational models, such as ordinary differential equations or agent-based models, can be employed to simulate the kinetics of complement activation pathways and metabolic processes within different cell types and tissues. By integrating experimental data on complement protein concentrations, metabolic fluxes, and cellular responses, these models can provide mechanistic insights into how complement activation or metabolic pathway changes impact immune cell function, inflammation, and pathogen clearance. For example, a model can generate hypotheses of how complement-driven metabolic rewiring affects outputs such as IFN-γ, IL-1 family cytokines, or inflammasome-linked programs by linking known intracellular models modulating inflammatory cytokines to complement-driven rewiring models. Without a model, testing such hypotheses would be challenged by the combinatorial complexity of the myriads of possible ways in which these pathways may affect each other. Moreover, intracellular complement functions across both innate and adaptive immune cells, as well as in some non-immune cells, while studies of immunometabolism show that even closely related populations, such as different T-cell subsets, can have markedly different metabolic requirements and rely to different degrees on glycolysis and oxidative phosphorylation. Together, these observations suggest that complement-driven metabolic effects are likely to be cell-type specific, making it natural for a mathematical model to ask which mechanisms or parameter differences account for those distinct responses using for example, sensitivity analyses.

### Determining Windows of Treatment in Tissue Injury

Complement activation has the potential side effect of tissue damage (Mollnes and Fosse 1994; Kavanagh and Goodship 2010; Pires and Calado 2023). Laboratory and/or clinical data suggest that complement is dysregulated in various diseases associated with acute lung injury such as COVID-19 (Ma et al. 2021; Afzali et al. 2022), influenza, (Sun et al. 2013; Song et al. 2018), paraquat-induced lung injury (Sun et al. 2011), MERS-CoV (Hamed et al. 2021), SARS-CoV, (Gralinski et al. 2018), LPS-induced ARDS, (Ali et al. 2023) and intestinal ischemia/reperfusion-induced lung injury (Hu et al. 2014). Clinical trials targeting complement in COVID-19 showed promising results, although many failed to improve the outcome due to possible confounding variables (Afzali et al. 2022). Timing, dosing, route of administration, pharmacokinetics, and target-level of complement therapeutics are key considerations. As previously mentioned, although complement therapeutics are administered systemically, their effects on local complement activation remain poorly understood. One potential problem for which mathematical modeling could prove beneficial is in predicting systemic drug concentrations which would result in an effective local complement effect (*e.g.,* using PK/PD). Treatment given too early with a complement-inhibitor could potentially impair proper clearance of pathogens and/or further predispose a patient to secondary infections. In addition to timing, other fundamental questions such as the level of the complement system (*e.g*., targeting total C3 or C5 instead of at the level of anaphylatoxins) or route of administration are key considerations in targeting complement. Getting complement activation “just right” is a complex optimization problem and drug efficacy can be obscured by the system’s complexity. Consider, for example, a disease where the MAC is ineffective against a pathogen (*e.g*., one of many Gram-positive bacteria, which are believed to be resistant to MAC-mediated killing). One might theorize that blocking C5 cleavage (*e.g.,* with eculizumab) could improve the outcome of the disease from preventing deposition of MAC on host cells (and thus self-damage). However, blocking C5 cleavage might be detrimental since it also prevents the formation of C5a fragments crucial for recruiting neutrophils and monocytes from the vasculature. If this theoretical experiment shows no improvement, it is difficult to tell whether MAC or C5a signaling blockage is beneficial. This can be tested in computational experiments. For example, expanding one of the models mentioned above that include clearance of pathogens, one could explore the overall effects of blocking MAC alone, blocking C5a signaling, or blocking both. Experimentally, these are difficult to separate—especially in complex models of disease. For example, blocking C5 has (at a minimum) the effect of blocking MAC formation and C5a signaling via C5aR1. Furthermore, mathematical modeling and tools such as optimal control could provide insight into the effects of each pathway computationally (conserving time and resources) while accounting for their simultaneity and interactions and guide future experiments.

### The Interaction Between Evasive Pathogens and Complement Proteins

Due to the central role of the complement system in the killing of encapsulated bacteria and other pathogens, several pathogens have strategies to evade complement-mediated immunity. For example, the mold of the *Aspergillus* species actively secretes proteins that interact and degrade various components of the complement system (Behnsen et al. 2010) and has many other mechanisms to inhibit a host’s complement repertoire, including the acquisition of complement regulatory proteins and masking of cell wall components (Speth and Rambach 2012). Gram-negative bacteria (*e.g*., *K. pneumoniae*) have mechanisms that enable them to hide from a host’s complement through encapsulation (Doorduijn et al. 2016). Glycoproteins can also protect viruses from complement-mediated neutralization, and the entry of virions into host cells is often facilitated via opsonization by complement and mediated by complement receptors (Mellors et al. 2020). Thus, there is a continuing/ongoing and dynamic battle between the hosts’ complement arsenal and the pathogen, and the outcome of this battle is often a key determinant of the patient’s outcome. Mathematical modeling of this complex battle could lead to novel strategies to tilt the tables towards the host. For example, would inhibiting (and by how much) a molecule that degrades complement proteins have an overall effect on the outcome of the disease? Alternatively, one could test in a computational platform the spiking of complement proteins to overwhelm the pathogen’s arsenal. Due to the rapid turnover of complement activation fragments, experimentally these are non-trivial to time correctly. Simpler experiments to assess the biochemical interaction and relevant parameters between pathogen proteins and host complement proteins could then be leveraged into a more complex host–pathogen model to prioritize both timing and amount of complement-mediated interventions.

## Conclusions and Future Directions

Since the discovery of the complement system by Jules Bordet in 1895, our understanding of complement as a liver-derived, serum operative system has evolved to a multifunctional system that touches on almost all cell types and tissues. The majority of mathematical models that focus on complement consider only the canonical functions of complement as a predominantly serum-based operator whose primary role is microbial clearance, and the complement-evading mechanisms of pathogens are often ignored in these models. It will be crucial to include these mechanisms of the pathogen as an organism that has its own arsenal to fight the complement system. Moreover, many of the parameters in such models are not experimentally derived but rather obtained using mathematical techniques. Thus, the potential of mathematical modeling of complement for optimal control, mechanism discovery, and experimental design remain limited. We argue that even with unidentified parameter values, theoretical mathematical modeling can offer insight that is rationally difficult to derive. Moreover, the advantage of having a structurally defined mathematical model is that the experimental results, whether positive or negative (in the sense of hypotheses testing) can be incorporated to refine the model and make predictive capacity more accurate. In addition, many biological quantities are difficult, if not impossible, to measure directly in the lab. Mathematical models can help estimate these hidden quantities and identify potential correlations or mechanisms that may not be obvious from direct observation of the data alone. 

Several challenges lie ahead. Many models’ timescale is also at the biochemical reaction scale (seconds to minutes), ignoring the cumulative effects of complement activity that lead to observable phenotype changes at the tissue level. Second, the effects of complement on physiology are largely ignored in these models. Third, while 18 of the 19 models covered in this review are explicitly defined (via formal mathematical equations), only 7 of these models have publicly available code. Given the size and complexity of most of these models, the lack of code availability is a large hurdle to their general use, especially due to the need to couple them with other types of models that are complex and able to handle spatial dynamics (*e.g*., hybrid ODE/PDE models and agent-based models of infections). Although several of the reviewed studies include components that could be considered part of a broader uncertainty-quantification workflow, such as local or global sensitivity analysis and parameter estimation, none of the models discussed here present a formal uncertainty quantification framework. In particular, we did not find studies that explicitly address structural or practical identifiability, report confidence intervals or posterior distributions for parameters, or propagate parameter uncertainty to generate uncertainty bands on model predictions. Consequently, the extent to which these models are identifiable, and how parameter uncertainty affects their predictive conclusions, often remains unclear. Finally, only a handful of the current mathematical models (3 out of 19, as of this writing) incorporate continuous spatial dynamics despite the importance of such dynamics in the open questions surveyed in the previous section. In light of this, there are many opportunities for spatial models to have impact.

Many of the models reviewed are highly complex and involve many parameters. While they have made important contributions to our understanding of complement biology, there is a clear need for a more structured modeling workflow that addresses key aspects of the modeling process, including model selection, parameter estimation using global search algorithms, uncertainty quantification, and parameter identifiability. In addition, model reproducibility remains an important issue. Not all studies make their full model implementation publicly available through a repository or accessible code archive, which can limit reproducibility, independent validation, and reuse of these models by other researchers. One potential future direction is to apply techniques of model reduction to alleviate some of the issues regarding large parameter spaces. We acknowledge that this is especially the case for modeling problems proposed that would require bridging together two already complex models (*e.g*. coagulation and complement), as this would make the uncertainty and identifiability an even more complex challenge. Another significant challenge is data availability and reducing the complexity of the biological problem at hand into manageable pieces. Many of the problems proposed in this manuscript will require significant new efforts in terms of data generation and validation to become sufficiently credible. The field of complement biology has evolved beyond structural biology to include more therapeutic and systems biology approaches in recent years**.** This will likely result in data generation that can be used to build models with higher fidelity and utility. There have been significant breakthroughs in complement therapeutics in rare diseases such as CHAPLE disease, paroxysmal nocturnal hemoglobinuria, and myasthenia gravis. Despite promising evidence, complement breakthroughs remain relatively rare, especially considering this system’s broad involvement and its prominent role in various diseases. As the field of complement-based therapeutics is nascent (the first complement drug was approved in 2007), it provides a great opportunity for mathematicians and systems biologists to apply integrative approaches toward discovering new interventions.
